# Supplementary material for: Risk factors for sacrococcygeal pilonidal sinus: a systematic review and meta-analysis supplemented by genetic causal assessment
Source: Front Surg. 2026 Jan 7;12:1718589. doi: 10.3389/fsurg.2025.1718589 (PMC12819706; doi:10.3389/fsurg.2025.1718589)
Supplement: Supplementary file 2 [file Datasheet2.zip › Supplementary Data 2/MR_pipeline_p5e-8/leaveone_plots/ukb-d-1747_4.finngen_R12_L12_PILONIDALCYST.pdf]

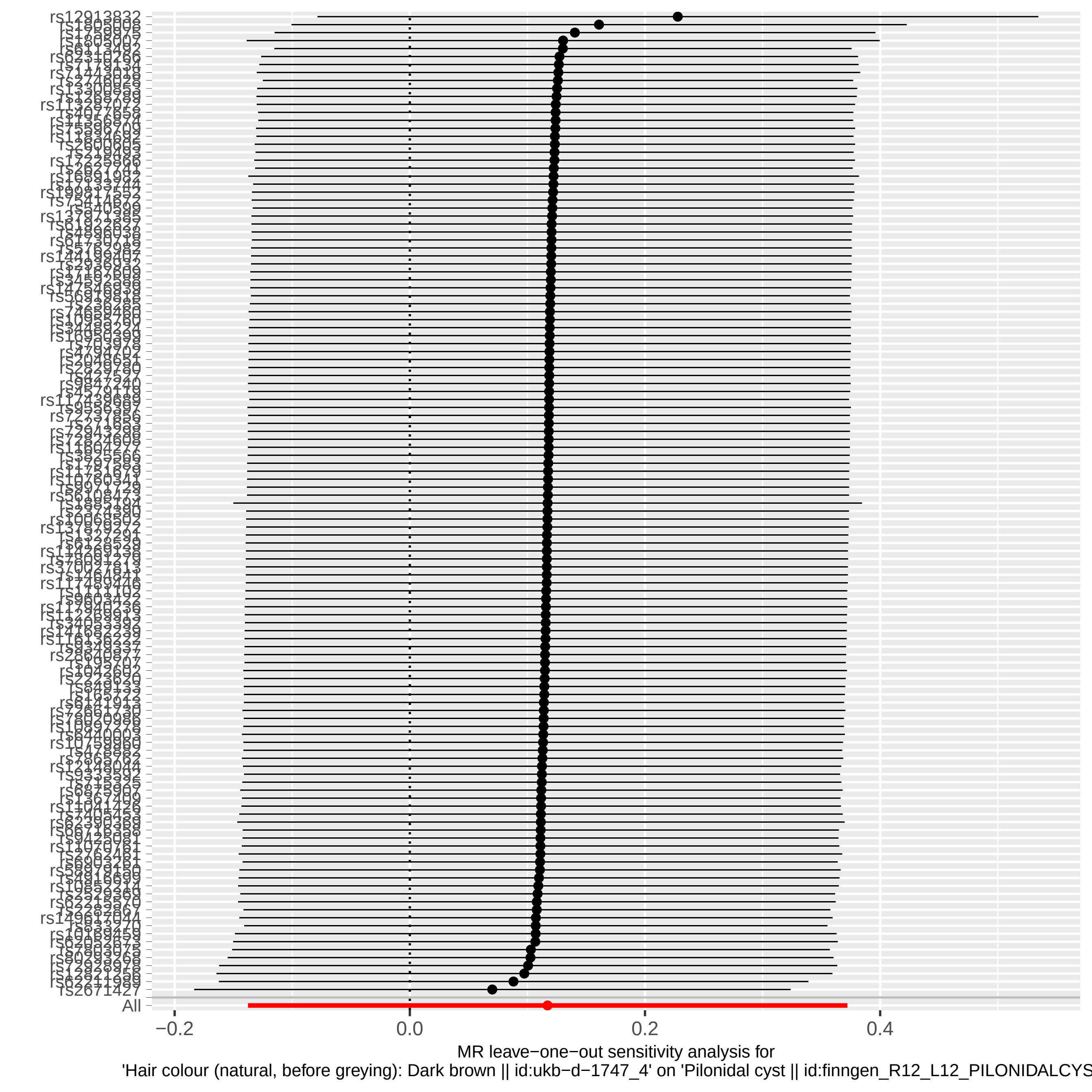
rs12913832
rs2671427
All
−0.2
0.0
0.2
0.4
MR leave−one−out sensitivity analysis for
'Hair colour (natural, before greying): Dark brown || id:ukb−d−1747_4' on 'Pilonidal cyst || id:finngen_R12_L12_PILONIDALCYS
